# Introducing automation to the molecular diagnosis of *Trypanosoma cruzi* infection: A comparative study of sample treatments, DNA extraction methods and real-time PCR assays

**DOI:** 10.1371/journal.pone.0195738

**Published:** 2018-04-17

**Authors:** Alba Abras, Cristina Ballart, Teresa Llovet, Carme Roig, Cristina Gutiérrez, Silvia Tebar, Pere Berenguer, María-Jesús Pinazo, Elizabeth Posada, Joaquim Gascón, Alejandro G. Schijman, Montserrat Gállego, Carmen Muñoz

**Affiliations:** 1 Secció de Parasitologia, Departament de Biologia, Sanitat i Medi Ambient, Facultat de Farmàcia i Ciències de l’Alimentació, Universitat de Barcelona, Barcelona, Spain; 2 ISGlobal, Barcelona Centre for International Health Research (CRESIB), Hospital Clínic–Universitat de Barcelona, Barcelona, Spain; 3 Laboratori d’Ictiologia Genètica, Departament de Biologia, Universitat de Girona, Girona, Spain; 4 Servei de Microbiologia, Hospital de la Santa Creu i Sant Pau, Barcelona, Spain; 5 Departament de Genètica i Microbiologia, Universitat Autònoma de Barcelona, Cerdanyola del Vallès, Spain; 6 Laboratorio de Biología Molecular de la Enfermedad de Chagas (LaBMECh), Instituto de Investigaciones en Ingeniería Genética y Biología Molecular “Dr. Héctor N. Torres” (INGEBI-CONICET), Buenos Aires, Argentina; 7 Institut d’Investigació Biomèdica Sant Pau (IIB Sant Pau), Hospital de la Santa Creu i Sant Pau, Barcelona, Spain; Oklahoma State University, UNITED STATES

## Abstract

**Background:**

Polymerase chain reaction (PCR) has become a useful tool for the diagnosis of *Trypanosoma cruzi* infection. The development of automated DNA extraction methodologies and PCR systems is an important step toward the standardization of protocols in routine diagnosis. To date, there are only two commercially available Real-Time PCR assays for the routine laboratory detection of *T*. *cruzi* DNA in clinical samples: TCRUZIDNA.CE (Diagnostic Bioprobes Srl) and RealCycler CHAG (Progenie Molecular). Our aim was to evaluate the RealCycler CHAG assay taking into account the whole process.

**Methodology/Principal findings:**

We assessed the usefulness of an automated DNA extraction system based on magnetic particles (EZ1 Virus Mini Kit v2.0, Qiagen) combined with a commercially available Real-Time PCR assay targeting satellite DNA (SatDNA) of *T*. *cruzi* (RealCycler CHAG), a methodology used for routine diagnosis in our hospital. It was compared with a well-known strategy combining a commercial DNA isolation kit based on silica columns (High Pure PCR Template Preparation Kit, Roche Diagnostics) with an *in-house* Real-Time PCR targeting SatDNA. The results of the two methodologies were in almost perfect agreement, indicating they can be used interchangeably. However, when variations in protocol factors were applied (sample treatment, extraction method and Real-Time PCR), the results were less convincing. A comprehensive fine-tuning of the whole procedure is the key to successful results. Guanidine EDTA-blood (GEB) samples are not suitable for DNA extraction based on magnetic particles due to inhibition, at least when samples are not processed immediately.

**Conclusions/Significance:**

This is the first study to evaluate the RealCycler CHAG assay taking into account the overall process, including three variables (sample treatment, extraction method and Real-Time PCR). Our findings may contribute to the harmonization of protocols between laboratories and to a wider application of Real-Time PCR in molecular diagnostic laboratories associated with health centers.

## Introduction

Chagas disease, a parasitic infection caused by the protozoan *Trypanosoma cruzi*, is endemic in 21 countries of Latin America, with approximately six million people affected [1]. Migratory flows have expanded Chagas disease worldwide, especially since the beginning of 2000, and the disease has emerged in non-endemic countries of North America, Europe and the Western Pacific Region [2,3].

In endemic settings the parasite is mainly transmitted by blood-sucking triatomine bugs [4,5], whereas in areas without vector-borne exposure the risk of developing *T*. *cruzi* infection arises from congenital transmission, blood transfusion, organ transplant, and laboratory accidents [6,7]. The disease has two stages: acute and chronic. Although both can be asymptomatic, the chronic phase is usually associated with cardiac and gastrointestinal disorders as well as low and intermittent parasitemia [8,9].

Several studies carried out in Spain and other parts of Europe have reported that screening for Chagas disease and early diagnosis is cost-effective [10–12]. However, a large number of patients in both endemic and non-endemic countries are still diagnosed late or not at all. Therefore, there is an urgent need to establish an efficient diagnostic strategy to deal with *T*. *cruzi* infection [13].

The serological diagnosis for Chagas disease is often difficult to interpret and although the use of a single test has been recently proposed [14], there is still no reference standard [15]. Serological methods are currently widely used, especially in the chronic phase, and dozens of tests are commercially available [16–19]. Nevertheless, serological diagnosis has certain disadvantages: the persistence of positive results in chronically infected patients for years after treatment [20], the possibility of cross-reactions with other trypanosomatids like *Trypanosoma rangeli* or *Leishmania* spp. [21], and the transmission of passive antibodies from mother to newborn [22–24].

Molecular biology techniques, especially the polymerase chain reaction (PCR), have been proposed as useful tools for the diagnosis of *T*. *cruzi* infection [25,26]. Unlike the serology test, a positive PCR result confirms the presence of the parasite DNA. Moreover, the high sensitivity of PCR in comparison with classical parasitological techniques is particularly useful for an early diagnosis of congenital cases [27–29].

Molecular detection of *T*. *cruzi* is also important in the chronic phase, as it can detect the therapeutic failure of anti-parasitic treatments and parasite reactivation in patients with immunosuppression [30,31]. Furthermore, the PCR variant known as quantitative Real-Time PCR also quantifies the amplification product using intercalating dyes or labeled probes and standard curves of known parasite concentration [32]. The main problem with this approach stems from the lack of consensus among laboratories on the PCR strategies used.

The recent development of automated DNA extraction methodologies coupled to PCR systems is an important step toward protocol standardization, while also preventing contamination of samples and reagents due to human manipulation. Automated nucleic acid extraction systems are usually based on magnetic separation, a time-saving technology in comparison with silica column-based DNA extractions [33,34]. One of the most applied molecular protocols is the amplification of satellite DNA (SatDNA) of *T*. *cruzi* [35,36]. SatDNA is the most abundant repetitive sequence in the parasite nuclear genome and is composed of about 10^5^ copies of a 195 nucleotide repeat [37,38].

To date, there are only two commercially available Real-Time PCR assays for the diagnosis of *T*. *cruzi* infection: TCRUZIDNA.CE (Diagnostic Bioprobes Srl, Sesto San Giovanni, Italy) and RealCycler CHAG (Progenie Molecular, Valencia, Spain). Both amplify the SatDNA sequence of *T*. *cruzi*. Seiringer et al. [39] recently evaluated TCRUZIDNA.CE but RealCycler CHAG has not been assessed until now, and no studies have previously evaluated these *T*. *cruzi* Real-Time PCR assays combined with different DNA extraction methods.

The aim of the present study was to assess the usefulness of an automated DNA extraction system based on magnetic particles (EZ1 Virus Mini Kit v2.0, Qiagen, Hilden, Germany) combined with a commercially available Real-Time PCR assay that targets the SatDNA of *T*. *cruzi* (RealCycler CHAG), a methodology routinely used for *T*. *cruzi*-infection diagnosis in the Hospital de la Santa Creu i Sant Pau of Barcelona (Spain). This technique was compared with a well-known and widely used strategy combining a commercial DNA isolation kit based on silica columns (High Pure PCR Template Preparation Kit, Roche Diagnostics, Mannheim, Germany) with an *in-house* Real-Time PCR that also targets the parasite SatDNA sequence [36].

## Materials and methods

### Ethics statement

Approval was obtained from the participating centers, the Clinical Research Ethics Committee of the Hospital de la Santa Creu i Sant Pau, the Ethics Review Committee of the Hospital Clínic and the Research Ethics Committee of the Universitat de Barcelona. All samples were anonymized before being evaluated and included in the study.

### Samples and population

A total of 123 blood samples were used in this study. Samples were collected from Hospital de la Santa Creu i Sant Pau and Hospital Clínic of Barcelona (Spain) during the period from January 2013 to March 2017. EDTA-blood (EB) and EDTA-blood mixed with an equal volume of guanidine hydrochloride solution 6 M (GEB) were obtained. Since the samples in the study were obtained retrospectively, in some cases only one of the two options, EB or GEB, was available. EB samples were stored at -40°C and GEB samples at 4°C until the subsequent analysis. Sample distribution was as follows: 19 from non-infected newborns from chagasic mothers, 5 from non-chagasic adults from endemic countries, 12 from non-chagasic individuals from non-endemic countries, 65 from non-treated chronic chagasic patients, and 22 seronegative GEB samples experimentally spiked with cultured epimastigotes of *T*. *cruzi* stocks as well as non-spiked GEB samples. Sample results were defined in the following way: non-infected newborns from chagasic mothers were followed up by serology until their negativization within the first year of life, non-chagasic adults were those with negative serology, and chronic chagasic patients were serologically positive by at least two different immunological techniques.

### DNA extraction procedures

Two different methodologies were used for the DNA extraction.

(i) Silica gel columns–High Pure PCR Template Preparation Kit (Roche Diagnostics). The protocol is available online at https://lifescience.roche.com. DNA extraction was performed according to the manufacturer’s instructions. DNA was extracted from 200 μL of EB or 300 μL of GEB and eluted in 100 μL of the elution buffer supplied with the kit, as previously described [40]. The extracted DNA was stored at -40°C until its analysis.

(ii) Magnetic particles–EZ1 Virus Mini Kit v2.0 (Qiagen). The protocol is available online at https://www.qiagen.com. DNA extraction was performed according to the manufacturer’s instructions using the EZ1 Advanced automated system based on magnetic particles. DNA was extracted from 400 μL of EB or GEB samples and eluted in 60 μL of the AVE buffer supplied with the kit. The extracted DNA was stored at -40°C until its analysis. EB samples were pre-treated with an equal volume of the erythrocyte lysis buffer (EL) (Qiagen), and 400 μL of the mixture was then introduced to the EZ1 device. GEB samples were directly loaded into the system.

For quantification, standard curves were built using non-chagasic human EB and GEB spiked with cultured epimastigotes of the *T*. *cruzi* Maracay strain (TcI), giving a final concentration of 10^6^ parasite equivalents/mL (par. eq./mL). DNA from spiked blood used to construct the standard curve was extracted with the High Pure PCR Template Preparation Kit and EZ1 Virus Mini Kit v2.0, and 1/10 serial dilutions of the DNA extracted in total blood DNA extractions from non-chagasic individuals were carried out in order to obtain a panel of samples ranging from 10^5^ to 1 par. eq./mL.

### Real-Time PCR methods

Two Real-Time PCR procedures were performed.

(i) *In house* Real-Time PCR targeting the *T*. *cruzi* SatDNA. Five μL of the DNA eluates in a final volume of 20 μL were amplified in triplicate. The final concentrations were: 1 x FastStart Universal Probe Master (Rox) (Roche Diagnostics), 0.75 μM of each primer Cruzi 1 and Cruzi 2, 0.25 μM of the probe Cruzi 3, and 0.2 x TaqMan Human RNase P detection reagent (Applied Biosystems, Austin, TX) [36]. The reaction was carried out in an ABI7900 device (Applied Biosystems) and the amplification of the RNase P human gene was included as an internal amplification control (IAC) [36]. Amplification conditions were as follows: one step of 10 min at 95°C, and 40 cycles of 95°C for 15 s, and 58°C for 1 min [40]. A sample was considered valid when the RNase P human gene was efficiently amplified with a cycle threshold value (Ct) ≤29, which was established with the Tukey criterion to detect outliers [41,42]. Samples were classified as inhibited when the IAC gave negative results or the Ct was >29. A sample was considered positive when the Ct of the target was ≤40 in at least one of the three replicates.

(ii) RealCycler CHAG (Progenie Molecular). The test was carried out according to the manufacturer’s instructions using the SmartCycler Automated Real-Time PCR system (Cepheid, Sunnyvale, CA). The kit includes the CHAG AmpliMix, which contains all the reagents necessary for the amplification as well as the IAC. The Real-Time PCR procedure was performed in 25 μL reaction volume containing 7.5 μL of eluted DNA and a single replica per sample was amplified. According to the manufacturer’s specifications, amplification conditions were as follows: one step of 15 min at 95°C, and 45 cycles of 95°C for 15 s, 60°C for 30 s, and 72°C for 30 s. A sample was considered valid when the IAC was amplified with a Ct ≤35 and inhibited when the criterion was not fulfilled. A sample was considered positive when the Ct of the target was ≤40.

### Experimental procedures

Sample treatments (EB and GEB), DNA extraction methods (silica columns and magnetic particles), and Real-Time PCR procedures (*In house* Real-Time PCR and RealCycler CHAG) were combined to create eight different methodological protocols for assessment (Table 1).

**Table 1:** *Trypanosoma cruzi* diagnostic Real-Time PCR results of samples analysed according to the protocols used in the study based on the combination of different sample treatments, DNA extraction methods and Real-Time PCR procedures.

Protocol code	Sample treatment	DNA extraction method	Real-Time PCR	N	Positive samples	Inhibited samples	N excluding inhibition
A (ref.)	GEB	Roche silica columns	*In house*	123	72	0	123
B	GEB	Roche silica columns	RealCycler	123	58	0	123
C	GEB	Qiagen magnetic particles	*In house*	62	8	35	27
D	GEB	Qiagen magnetic particles	RealCycler	62	25	17	45
E	EB	Roche silica columns	*In house*	25	11	0	25
F	EB	Roche silica columns	RealCycler	25	11	0	25
G	EB	Qiagen magnetic particles	*In house*	64	15	2	62
H	EB	Qiagen magnetic particles	RealCycler	64	15	0	64

Inhibited samples had negative results or a cycle threshold (Ct) value >29 for the internal amplification control in protocols using *in house* Real-Time PCR (A, C, E, and G), and negative results or Ct >35 in protocols using RealCycler CHAG (B, D, F, and H).

Ref.: reference standard protocol, EB: EDTA-blood, GEB: Guanidine EDTA-blood, N: number of samples.

The *in house* protocol A was considered the reference standard due to its validation in previous international studies [26,42] and the commercialized protocol H is used for *T*. *cruzi* routine diagnosis in the Hospital de la Santa Creu i Sant Pau.

### Data analysis

Taken as the reference standard, protocol A was compared with all the others (B to H), resulting in seven possible combinations (A-B, A-C, A-D, A-E, A-F, A-G, and A-H). Protocols were also compared by focusing on only one variable of the process: sample treatments, DNA extraction methods or Real-Time PCR procedures. In this way, 12 combinations were created: A-E, B-F, C-G, and D-H comparing sample treatments; A-C, B-D, E-G, and F-H comparing DNA extraction methods; and A-B, C-D, E-F, and G-H comparing Real-Time PCR procedures. For the protocol comparisons, Cohen’s kappa coefficient (K), which describes the level of concordance between two tests relating the observed agreement and the agreement expected by chance, was calculated for each protocol combination created. The interpretation of K values was as follows: 0 to 0.20 indicates slight agreement, 0.21 to 0.40 fair agreement, 0.41 to 0.60 moderate agreement, 0.61 to 0.80 substantial agreement, and 0.8 to 1 almost perfect agreement [43]. Calculations were performed with the software EPIDAT 3.1, which is available online at http://www.sergas.es/Saude-publica.

Parasitic loads obtained from positive samples in protocols A and H were compared in a Bland-Altman difference plot in order to quantify the agreement between the two methods [44]. The Spearman rank correlation coefficient (r_s_), which describes the strength of a monotonic relationship between paired data, was also calculated [45]. Ranging from -1.0 to +1.0, values close to +1.0 indicate positive association whereas values close to -1.0 indicate negative association. The interpretation of the absolute value of r_s_ is as follows: 0 to 0.19 indicates a very weak relationship, 0.20 to 0.39 weak, 0.40 to 0.59 moderate, 0.60 to 0.79 strong, and 0.80 to 1.0 a very strong relationship.

## Results

A total of 123 blood samples were analyzed. Since this is a retrospective study, the number of samples analyzed with each protocol is not identical (Table 1).

The results of the comparison between the reference protocol A and the others are summarized in Table 2. K results are shown including and excluding inhibited samples. In protocols using GEB samples a high level of inhibition was found when the DNA extraction method based on magnetic particles was used, as shown in protocols C and D with 56.5% and 27.4% of inhibition, respectively (Table 1). In the other cases, protocols did not present inhibition, with the exception of protocol G (EB, magnetic particles and *in house* Real-Time PCR), which yielded two inhibited samples (3.1%).

**Table 2:** Cohen’s kappa coefficient (K) results for the comparison between the reference protocol A and the others. K values are shown including and excluding inhibited samples.

Protocols	N	K value (95% CI)	Positive samples	Inhibited samples	N excluding inhibition	Discordant samples excluding inhibition	K value excluding inhibited samples (95% CI)
A-B	123	0.79 (0.68–0.9)	59	0	123	13	0.79 (0.68–0.9)
A-C	62	0.17 (0.08–0.26)	8	35	27	8	0.45 (0.18–0.72)
A-D	62	0.29 (0.14–0.44)	24	17	45	10	0.53 (0.3–0.77)
A-E	25	0.92 (0.77–1)	11	0	25	1	0.92 (0.77–1)
A-F	25	0.76 (0.5–1)	10	0	25	3	0.76 (0.5–1)
A-G	64	0.92 (0.81–1)	15	2	62	0	1
A-H	64	1	15	0	64	0	1

N: number of samples assessed in parallel by both protocols compared. Positive samples: number of samples with a positive result for both protocols. Inhibited samples: number of samples with an invalid result for the internal amplification control (IAC) in at least one of the two protocols compared. CI: confidence interval.

The number of discordant results between protocols ranged from 1 to 13. The results obtained with these samples are listed in Table 3.

**Table 3:** *Trypanosoma cruzi* diagnostic Real-Time PCR results obtained in the eight protocols assessed for the 22 discordant samples.

Sample ID	Protocols^a^							
	A	B	C	D	E	F	G	H
S1	N	N	N	N	N	P (40) 1/1	N	N
S2	P (32.2) 3/3	P (32.2) 1/1	P (29) 3/3	P (31.9) 1/1	P (31.9) 3/3	N	P (32.9) 3/3	P (35) 1/1
S3	N	N	N	P (40) 1/1	N	N	N	N
S4	P (28.2) 3/3	P (30.5) 1/1	N	P (28.3) 1/1	P (36.2) 3/3	P (39.5) 1/1	P (25.8) 3/3	P (28.5) 1/1
S5	P (29.6) 3/3	P (30.6) 1/1	P (28.5) 3/3	P (31.2) 1/1	N	N	P (26.9) 3/3	P (30.6) 1/1
S6	P (35.1) 1/3	N	I	I	-	-	-	-
S7	P (32.2) 2/3	N	I	P (34.2) 1/1	-	-	-	-
S8	P (35.3) 1/3	N	I	N	-	-	-	-
S9	P (29.9) 3/3	P (34.1) 1/1	N	P (32.2) 1/1	-	-	-	-
S10	P (36.4) 1/3	N	N	N	-	-	-	-
S11	P (35.5) 1/3	N	I	N	-	-	-	-
S12	P (29.4) 3/3	P (31.8) 1/1	N	P (31.2) 1/1	-	-	-	-
S13	P (35.6) 1/3	N	N	N	-	-	-	-
S14	P (35) 1/3	N	I	I	-	-	-	-
S15	P (36.3) 1/3	P (38.4) 1/1	I	N	-	-	-	-
S16	P (35.9) 1/3	N	N	N	-	-	-	-
S17	P (33.4) 2/3	N	I	I	-	-	-	-
S18	P (34.9) 2/3	N	I	N	-	-	-	-
S19	P (33.7) 1/3	N	I	I	-	-	-	-
S20	P (34.6) 1/3	P (39.7) 1/1	I	N	-	-	-	-
S21	P (35.4) 1/3	N	N	I	-	-	-	-
S22	P (35.4) 1/3	N	N	N	-	-	-	-

Protocols A, C, E, and G used the *in house* Real-Time PCR with three replicates per sample. Protocols B, D, F, and H used RealCycler CHAG Real-Time PCR with a single replica amplification per sample.

^a^Cycle threshold (Ct) results are shown in parentheses. In case of multiple positive replicates, the mean of the Cts obtained is indicated. The number of positive replicates is expressed as a fraction below.

P: positive, N: negative, I: inhibited.

**Fig 1:**
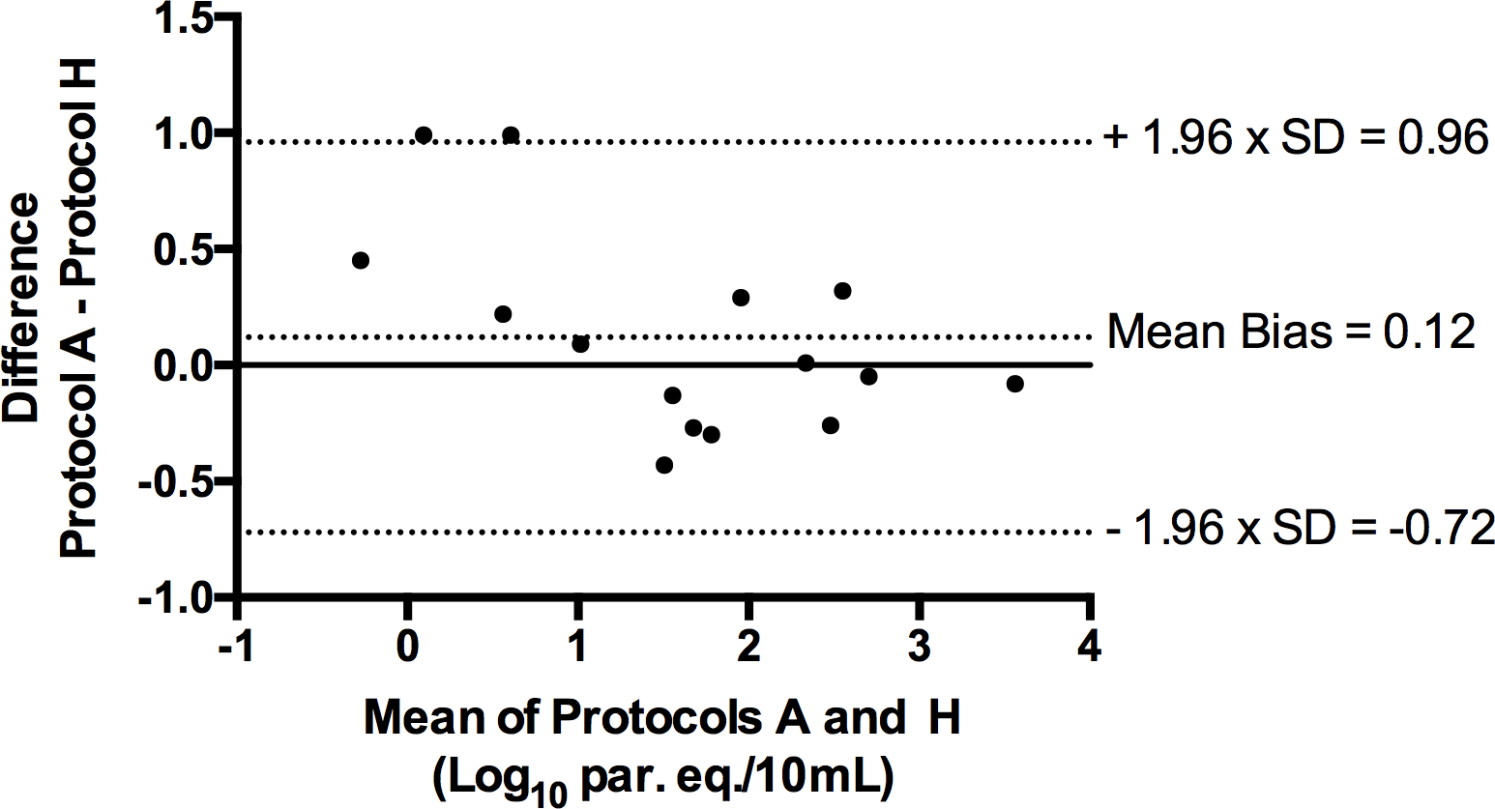
Degree of agreement between protocols A and H based on a Bland-Altman plot of the nine samples with quantifiable results in both methods. Each sample is represented by plotting the mean of the measurements obtained in protocols A and H on the x-axis and the difference of the same two values on the y-axis. SD: standard deviation. Log_10_ par. eq./10 mL: logarithmic values of parasite equivalents in 10 mL of blood.

Protocols A (reference standard) and H (routine diagnosis in our hospital) gave matching qualitative results for the 64 samples analysed (K = 1), 15 of which were positive. These protocols also underwent a quantitative evaluation (Table 4). The level of agreement between the positive samples in both methods is represented by a Bland-Altman difference plot (Fig 1). The mean bias was determined as 0.12 Log_10_ par. eq./10 mL, indicating a systematic bias of 0.7-fold parasite equivalents per 10 mL between methods. The bias was in both directions and did not differ in relation to the magnitude of the parasitic loads. However, samples with the highest bias yielded very low parasitic loads (samples S2 and S11 in Table 4). Limits of agreement were also included in the plot expressed as bias ± 1.96 x standard deviation (SD). The SD was 0.43, the lower limit of agreement with a 95% confidence interval (CI) was -0.72 Log_10_ par. eq./10 mL and the upper one was 0.96 Log_10_ par. eq./10 mL. The Spearman rank correlation coefficient was r_s_ = 0.97, which indicates very strong and positive correlation between protocols.

**Table 4:** Quantitative evaluation (parasitic load) of the 15 samples positive in protocols A (reference standard) and H (Hospital de la Santa Creu i Sant Pau routine diagnosis).

	Protocol A		Protocol H	
Sample ID	Par. eq./mL	Log_10_ Par. eq./10 mL	Par. eq./mL	Log_10_ Par. eq./10 mL
S1	0.47	0.67	0.28	0.45
S2	1.26	1.1	0.13	0.11
S3	51.80	2.71	24.79	2.39
S4	4.30	1.63	8.50	1.93
S5	21.90	2.34	21.55	2.33
S6	47.80	2.68	54.09	2.73
S7	12.49	2.1	6.43	1.81
S8	22.39	2.35	40.88	2.61
S9	331.00	3.52	395.66	3.6
S10	0.09	-0.05	0.03	-0.5
S11	0.39	0.59	0.04	-0.4
S12	1.94	1.29	5.21	1.72
S13	3.09	1.49	4.18	1.62
S14	1.14	1.06	0.94	0.97
S15	3.44	1.54	6.43	1.81

Parasitic loads are expressed in parasite equivalents in one mL of blood (Par. eq./mL) and in logarithms of the parasite equivalents in 10 mL of blood (Log_10_ Par. eq./10 mL).

Protocols were also compared taking into account only one variable of the procedure (Tables 5–7). K values and positive and inhibited sample results are shown for the following variables: (i) sample treatment (Table 5); (ii) DNA extraction method (Table 6); and (iii) Real-Time PCR procedures (Table 7). Modifications in one of the three variables resulted in changes in the K values, the number of inhibited samples and the percentage of discordant results obtained.

**Table 5:** Cohen’s kappa coefficient (K) results for the comparison between protocols with the sample treatment as the only variable. K values are shown including and excluding inhibited samples.

Protocols	N	K value (95% CI)	Positive samples	Inhibited samples	N excluding inhibition	Discordant samples excluding inhibition	K value excluding inhibited samples (95% CI)
A-E	25	0.92 (0.77–1)	11	0	25	1	0.92 (0.77–1)
B-F	25	0.76 (0.5–1)	10	0	25	3	0.76 (0.5–1)
C-G	25	0.45 (0.25–0.65)	5	8	17	1	0.87 (0.61–1)
D-H	25	0.55 (0.32–1)	7	6	19	1	0.89 (0.68–1)

N: number of samples assessed in parallel by both protocols compared. Positive samples: number of samples with a positive result for both protocols. Inhibited samples: number of samples with an invalid result for the internal amplification control (IAC) in at least one of the two protocols compared. CI: confidence interval.

**Table 6:** Cohen’s kappa coefficient (K) results for the comparison between protocols with the DNA extraction system as the only variable. K values are shown including and excluding inhibited samples.

Protocols	N	K value (95% CI)	Positive samples	Inhibited samples	N excluding inhibition	Discordant samples excluding inhibition	K value excluding inhibited samples (95% CI)
A-C	62	0.17 (0.08–0.26)	8	35	27	8	0.45 (0.18–0.72)
B-D	62	0.46 (0.31–0.61)	23	17	45	4	0.82 (0.65–0.99)
E-G	25	0.92 (0.77–1)	11	0	25	1	0.92 (0.77–1)
F-H	25	0.76 (0.5–1)	10	0	25	3	0.76 (0.5–1)

N: number of samples assessed in parallel by both protocols compared. Positive samples: number of samples with a positive result for both protocols. Inhibited samples: number of samples with an invalid result for the internal amplification control (IAC) in at least one of the two protocols compared. CI: confidence interval.

**Table 7:** Cohen’s kappa coefficient (K) results for the comparison between protocols with the Real-Time PCR procedure as the only variable. K values are shown including and excluding inhibited samples.

Protocols	N	K value (95% CI)	Positive samples	Inhibited samples	N excluding inhibition	Discordant samples excluding inhibition	K value excluding inhibited samples (95% CI)
A-B	123	0.79 (0.68–0.9)	59	0	123	13^a^	0.79 (0.68–0.9)
C-D	62	0.44 (0.29–0.6)	8	35	27	4	0.68 (0.41–0.96)
E-F	25	0.84 (0.62–1)	10	0	25	2	0.84 (0.62–1)
G-H	64	0.92 (0.81–1)	15	2	62	0	1

^a^None of these samples were positive in all three replicates.

N: number of samples assessed in parallel by both protocols compared. Positive samples: number of samples with a positive result for both protocols. Inhibited samples: number of samples with an invalid result for the internal amplification control (IAC) in at least one of the two protocols compared. CI: confidence interval.

## Discussion

New strategies for Chagas disease diagnosis have been proposed with the introduction of commercially available tests. Their implementation should bring about a considerable improvement in the diagnosis of *T*. *cruzi* infection and lead to the standardization of protocols between laboratories. Regarding molecular tests, to our knowledge only two Real-Time PCR assays (TCRUZIDNA.CE and RealCycler CHAG) are commercially available for the routine laboratory detection of *T*. *cruzi* DNA in clinical samples, both developed in Europe. Seiringer et al. [39] have recently assessed TCRUZIDNA.CE, which showed high sensitivity and specificity when compared with widely used PCR and Real-Time PCR strategies [26,46–48]. The study, performed in Italy, evaluated the PCR amplification but not the sample treatment or the extraction method. On the other hand, the RealCycler CHAG has not yet been assessed by comparison with other PCR assays, as recommended [30]. Thus, the aim of the work presented here was to assess this Real-Time PCR system taking into account the sample treatment and DNA extraction method, and is therefore the first study to evaluate the overall process including these three variables.

First of all, we created a panel of eight different protocols (Table 1) through the combination of three factors (sample treatments, DNA extraction methods, and Real-Time PCR procedures) and compared each one with the protocol considered the reference standard (protocol A in Table 1). All combinations reached a high concordance level except when the standard was compared with protocols starting from GEB and using the DNA extraction method based on magnetic particles (protocols C and D), which showed a high level of inhibition (Tables 1 and 2). A possible explanation is that the magnetic particle DNA isolation system was unable to completely eliminate the guanidine hydrochloride during the extraction process and the residual solution in the extracted DNA inhibited the PCR [35,49]. An alternative explanation is an excess of guanidine hydrochloride, given that some buffers of the kit reagent cartridges already contain guanidine salts. Indeed, all extracted DNA from inhibited samples showed a yellowish coloration, which was an early indication that the process had not worked properly. Moreover, it was noted that inhibition was related to the time elapsed between the sample treatment with guanidine and the DNA extraction: all inhibited samples were processed at least six months before being mixed with guanidine. In contrast, no inhibition was observed when DNA extraction occurred within the first week after the guanidine treatment. In the EB samples practically no inhibition occurred; the two exceptional samples (out of 64) had undergone magnetic particle DNA extraction (protocol G), and there was probably a residual pool of magnetic particles in the extracted DNA [50].

In this study, the RNase P human gene was used as an IAC to detect inhibition of the *in house* Real-Time PCR, as previously described by Pirón et al. [36]. However, we observed that patients with leukopenia or neutropenia showed later Ct values in the Real-Time PCR than immunocompetent individuals, rather than inhibition, as a consequence of their immunological condition. This should therefore be born in mind when hematological patients are included in studies of this kind. In fact, Duffy et al. [40] suggested the use of a heterologous extrinsic IAC, which has subsequently been taken up in other studies [42,51,52]. Likewise, the RealCycler assay includes this kind of control among the reagents supplied with the kit.

Once the inhibited samples were excluded from the analysis, discordant results appeared with regard to the extraction method and the number of replicates amplified in the Real-Time PCR. When comparing the reference protocol with those using GEB and magnetic particle DNA extraction (Table 2), the results suggest that silica column-based DNA extraction works more efficiently than the magnetic particle system with GEB samples. In contrast, the two DNA extraction methodologies presented similar results with EB samples (Table 6). Taking into account the number of replicates amplified in the Real-Time PCR, protocols using the RealCycler Real-Time PCR included only one reaction of amplification per sample instead of three, thus reducing the possibility of obtaining a positive result (Table 3). An increase in the number of replicates in these protocols would probably reduce conflicting results. Otherwise there were no important differences in results between protocols when comparing Real-Time PCRs (Table 7), which could be expected since both target the parasite SatDNA and probably use the same primer and probe sequences. However, the commercial formulation of the RealCycler CHAG assay is not known.

The results obtained highlight the importance of fine-tuning the overall process, including sample treatment, DNA extraction method and Real-Time PCR, for the successful diagnosis of *T*. *cruzi* infection. The reference standard protocol (A) and the one used for routine diagnosis in our hospital (H) were in perfect concordance, but the results were less convincing when factor variations were introduced (Table 2).

The quantitative analysis was only performed for protocols A and H because their comparison was the only one to show perfect agreement and no inhibition. The 15 samples positive with both protocols belonged to chronic patients, which probably explains why five samples had very low parasitic loads (samples S1, S2, S10, S11, and S14 in Table 4). The number of samples in our study was a limiting factor for the analysis of the results. According to Bland and Altman [44], some lack of agreement between different methods of measurement is inevitable, therefore what is important is the amount by which methods disagree. Based on this assumption, a Bland-Altman plot was constructed (Fig 1). As can be seen from the plot, the bias is not statistically significant, so protocols A and H were determined as equivalent and interchangeable [53].

## Conclusions

The reference protocol (A) and the one used for routine molecular diagnosis of *T*. *cruzi* DNA in our hospital (H) were in perfect concordance and both tests can be used interchangeably. Therefore, protocol H (EB samples, EZ1 Virus Mini Kit v2.0 and the RealCycler CHAG) is a good option for the routine diagnosis of *T*. *cruzi* infection. The combination of these two assays allows results to be obtained in just over two hours, in a straightforward way and with minimum handling, which indicates its suitability for incorporation in hospital-associated laboratories. When variations in protocol factors were applied, the results were less convincing, which highlights that the overall process needs to be fine-tuned to obtain good results. The large number of inhibited GEB samples detected indicates they are not suitable for the DNA extraction method based on magnetic particles, at least when samples are not processed immediately.
